# Simultaneous improvement and genetic dissection of drought and submergence tolerances in rice (*Oryza sativa* L.) by selective introgression

**DOI:** 10.3389/fpls.2023.1134450

**Published:** 2023-04-18

**Authors:** Chaopu Zhang, Min Li, Jessica Domingo Rey, Ting Feng, Renee Lafitte, Tianqing Zheng, Yamei Lv, Fengcai Wu, Binying Fu, Jianlong Xu, Fan Zhang, Wei Zeng, Erbao Liu, Jauhar Ali, Wensheng Wang, Zhikang Li

**Affiliations:** ^1^ College of Agronomy, Anhui Agricultural University, Hefei, China; ^2^ International Rice Research Institute, Manila, Philippines; ^3^ Institute of Biology, College of Science, UP Diliman, Philippines; ^4^ Institute of Crop Sciences, Chinese Academy of Agricultural Sciences, Beijing, China; ^5^ Hainan Yazhou Bay Seed Lab/National Nanfan Research Institute (Sanya), Chinese Academy of Agricultural Sciences, Sanya, China; ^6^ Shenzhen Branch, Guangdong Laboratory for Lingnan Modern Agriculture, Genome Analysis Laboratory of the Ministry of Agriculture, Agricultural Genomics Institute at Shenzhen, Chinese Academy of Agricultural Sciences, Shenzhen, China

**Keywords:** drought tolerance, submergence tolerance, rice, selective introgression, loss of heterozygosity

## Abstract

**Introduction:**

Drought and submergence are contrasting abiotic stresses that often occur in the same rice crop season and cause complete crop failure in many rain-fed lowland areas of Asia.

**Methods:**

To develop rice varieties with good tolerances to drought and submergence, 260 introgression lines (ILs) selected for drought tolerance (DT) from nine BC_2_ populations were screened for submergence tolerance (ST), resulting in 124 ILs with significantly improved ST.

**Results:**

Genetic characterization of the 260 ILs with DNA markers identified 59 DT quantitative trait loci (QTLs) and 68 ST QTLs with an average 55% of the identified QTLs associated with both DT and ST. Approximately 50% of the DT QTLs showed ‘epigenetic’ segregation with very high donor introgression and/or loss of heterozygosity (LOH). Detailed comparison of the ST QTLs identified in ILs selected only for ST with ST QTLs detected in the DT-ST selected ILs of the same populations revealed three groups of QTLs underlying the relationship between DT and ST in rice: a) QTLs with pleiotropic effects on both DT and ST; b) QTLs with opposite effects on DT and ST; and c) QTLs with independent effects on DT and ST. Combined evidence identified most likely candidate genes for eight major QTLs affecting both DT and ST. Moreover, group b QTLs were involved in the *Sub1*regulated pathway that were negatively associated with most group aQTLs.

**Discussion:**

These results were consistent with the current knowledge that DT and ST in rice are controlled by complex cross-talks between or among different phytohormone-mediated signaling pathways. Again, the results demonstrated that the strategy of selective introgression was powerful and efficient for simultaneous improvement and genetic dissection of multiple complex traits, including DT and ST.

## Introduction

Rice is one of the most important cereals and staple food for most people in Asia. Rice is known for its adaptation to flooded conditions. Most rice varieties are highly sensitive to drought or water deficit, which is the most important factor limiting rice productivity in many rainfed areas of Asia (Pandey et al., 2000). However, most rice cultivars are also vulnerable to complete submergence for an extended period of time. This type of short-term flash floods of 1–2 weeks may occur at any stage of rice development and cause severe yield losses. This is particularly true in the large areas of Bangladesh, India, Cambodia and Vietnam in South and Southeast Asia. Thus, drought and submergence represent two opposite environmental stresses to rice crops, but they often occur at different times of the same rice crop season in many areas of the rainfed-lowland ecosystems of Asia. In some areas of Bangladesh and India, submergence often occurs in the early rice crop season, causing significant damages on crop establishment, whereas drought occurs more frequently in the late crop season, causing great yield losses by reducing fertility and grain filling of rice crops. Thus, plant breeders in these areas are facing a tremendous challenge to develop rice cultivars that are tolerant to both submergence and drought.

Rice varieties differ significantly for their submergence and drought tolerances (ST and DT) (Setter et al., 1997; Lafitte et al., 2006). Genetically, both DT and ST are under complex genetic control (Nandi et al., 1997; Sripongpangkul et al., 2000; Toojinda et al., 2003., Wang et al., 2015; Cui et al., 2018), though cases of major genes controlling ST or DT have been reported (Xu and Mackill, 1996; Benschop et al., 2005; Bernier et al., 2007). However, as separate research activities, these theoretical studies have not been applied by breeders to improving their breeding efficiencies in developing DT and ST varieties. Meanwhile, considerable progress has been made in developing lines tolerant to single abiotic stresses such as for drought, submergence, salinity, etc. either by BC breeding or by marker-assisted selection (MAS) (Ali et al., 2006; Lafitte et al., 2006; He et al., 2010; Yasmin et al., 2014; Li and Ali, 2017). More recently, the strategy of selective introgression (SI) has been successfully applied for simultaneous improvement and genetic dissection of rice tolerances to single abiotic stresses such as drought (Li et al., 2005; Cui et al., 2018), submergence (Wang et al., 2015), low temperature (Meng et al., 2013; Zhang et al., 2014; Liang et al., 2018), salt (Wang et al., 2013). However, few efforts have been taken to develop rice cultivars with significantly improved DT and ST in the same breeding programs. The primary reason appeared to be the lack of superior germplasm accessions with excellent DT and ST as donors of breeding efforts. Another reason was the apparent negative association between ST and DT in rice at the phenotypic level, as some rice lines such as FR13A with excellent ST tend to be highly sensitive to drought and most DT upland rice varieties tend to be highly susceptible to submergence (Lafitte et al., 2006; Wang et al., 2015). As a result, few rice varieties with excellent DT, ST, and superior yield potential have been developed despite this type of DT/ST rice cultivars are desperately needed by farmers in many rainfed areas of South Asia. Thus, developing superior rice varieties with good ST and DT remains a great challenge to rice breeders. To overcome this challenge, two important questions have to be answered. First, is it possible to develop rice varieties with greatly improved DT and ST genetically, and if so, where is the source of genetic variation for simultaneous improvement of DT and ST in rice? Second, what is the genetic basis for the observed phenotypic association between ST and DT in some rice accessions such as FR13A?

In this study, by characterizing genomewide responses of donor introgression in progenies selected first for DT followed by two rounds of selection for improved ST from nine BC_2_ populations, our results provided answers to the two questions and suggested effective breeding strategies for improving either and/or DT and ST of rice in future.

## Materials and methods

### Development of ILs tolerant to both drought and submergence

The materials used in this study included nine BC_2_F_2_ populations derived from crosses between two elite *Xian* (*indica*) recipients, (IR64 from Philippines and Teqing from China), and seven diverse donors from six countries (**Supplementary Table S1**). The donors included Shwe-Thwe-Yin-Hyv (STYH) from Myanmar, BR24 from Bangladesh, Type3 (a *Geng* or *japonica* variety) from India, Cisanggarung (Cis, a lowland *Xian* variety) from Indonesia, Binam (a *Basmati* landrace) from Iran, and OM1723 (a lowland *Xian* variety) from Vietnam and FR13A (the best ST *Xian* landrace) from India. The procedure of the BC breeding was described previously (Ali et al., 2006). Briefly, the two recipients were crossed with all donors to produce the F_1_s. The F_1_s were backcrossed with the recipients to produce the BC_1_F_1_s. Approximately 25 plants from each BC_1_F_1_ line were backcrossed with each recipient to produce ~25 BC_2_F_1_ lines. From each of the crosses, 25 BC_2_F_1_ lines were planted in the following season and seeds all 25 BC_2_F_1_ lines of each cross were bulk-harvested to form a single bulk BC_2_F_2_ population.

### Screening of DT

In the dry season (DS) of 2004, seeds from each BC_2_F_2_ population were sown on the seedling nursery with the parental lines and 208 25-day old seedlings from each population and the recipients were transplanted into a paddy field at the International Rice Research Institute (IRRI) experimental farm in the Philippines. Normal irrigation was applied until the peak tillering stage (30 days after transplanting) and then water was withheld until maturity, subjecting all plants to severe terminal drought at the reproductive stage. In addition, the bulk harvested seeds of each 9 BC_2_F_2_ population were directly sown into a five-row plot (with ~250 plants/plot) flanked by two rows of the recipients in the upland (aerobic) conditions in the IRRI upland facility, and then the fields was irrigated with flash water to allow seeds to germinate until the five-leaf age and then irrigated was withheld, subjecting all plants to chronic water stress from the seedling stage to maturity. Under both the lowland and upland drought stresses, the recipients, IR64 and Teqing were unable to produce any seeds, while some plants in each of the BC_2_F_2_ populations survived and were able to produce seeds, which were individually selected to produce respective BC_2_F_3_ lines.

### Progeny testing and screening for DT

All BC_2_F_3_ introgression lines (ILs) selected from the DT screening were progeny tested in the replicated experiments in the upland (stress) and normal irrigated lowland conditions in the coming wet season (WS) and dry season (DS) of 2005-2006 at IRRI. In the progeny testing, seeds of all selected BC_2_F_3_ ILs were sown in the seedling nursery in late May and 25-day old seedlings of each BC_2_F_3_ IL or their parents were transplanted into a three-row plot with 33 plants per plot at a spacing of 20 × 25 cm in the field. The plots were arranged with a complete randomized block design with three replications for each IL plus insertions of the recipient plots (IR64 and Teqing) in every 20 plots as checks in the field experiment. The same experimental design was used in the normal irrigated control. All selected ILs were shown to have improved DT based on the progeny testing (Lafitte et al., 2006).

### Screening of the BC_2_ Introgression lines (ILs) for Submergence Tolerance (ST)

In the following WS of 2005, all drought selected BC_2_F_3_ ILs were screened for their ST. Briefly, the evaluation of ST was performed in the 3m-deep-pond facility of IRRI. Seeds of each of the drought selected BC_2_F_3_ ILs, the recipients, and a susceptible check (IR42), were sown in a single row (~15 plants per row) on seedling nursery of the facility with two replications for each BC_2_F_3_ IL, recipients and IR42. The 30-day old seedlings were submerged under 1.5 m-deep water and maintained under the complete submergence for two weeks until the susceptible check (IR42) and recipients were killed. Water was then drained and the surviving/recovering plants of each BC_2_F_3_ IL were counted and converted into surviving or recovering percentage. In the following DS, seeds collected form all survived BC_2_F_4_ ILs from the submergence evaluation in the WS of 2006 were tested in the same way for their ST with two-week complete submergence. In the confirmation experiment, 5g of seeds for each selected BC_2_F_4_ IL were sown in a 75-cm long row with 15 cm between rows. At 30 days after sowing, the water level was increased up to 1.3 m and was maintained up to 14 days until the susceptible check IR42 and recipients were all dead. Then, the water was totally drained out of the pond. A IL was considered to be ST when its average surviving/recovering rate was greater than 50% in the BC_2_F_3_ screening and BC_2_F_4_ progeny testing under complete submergence in the DS and WS experiments.

### Genotyping experiments

**Figure 1:**
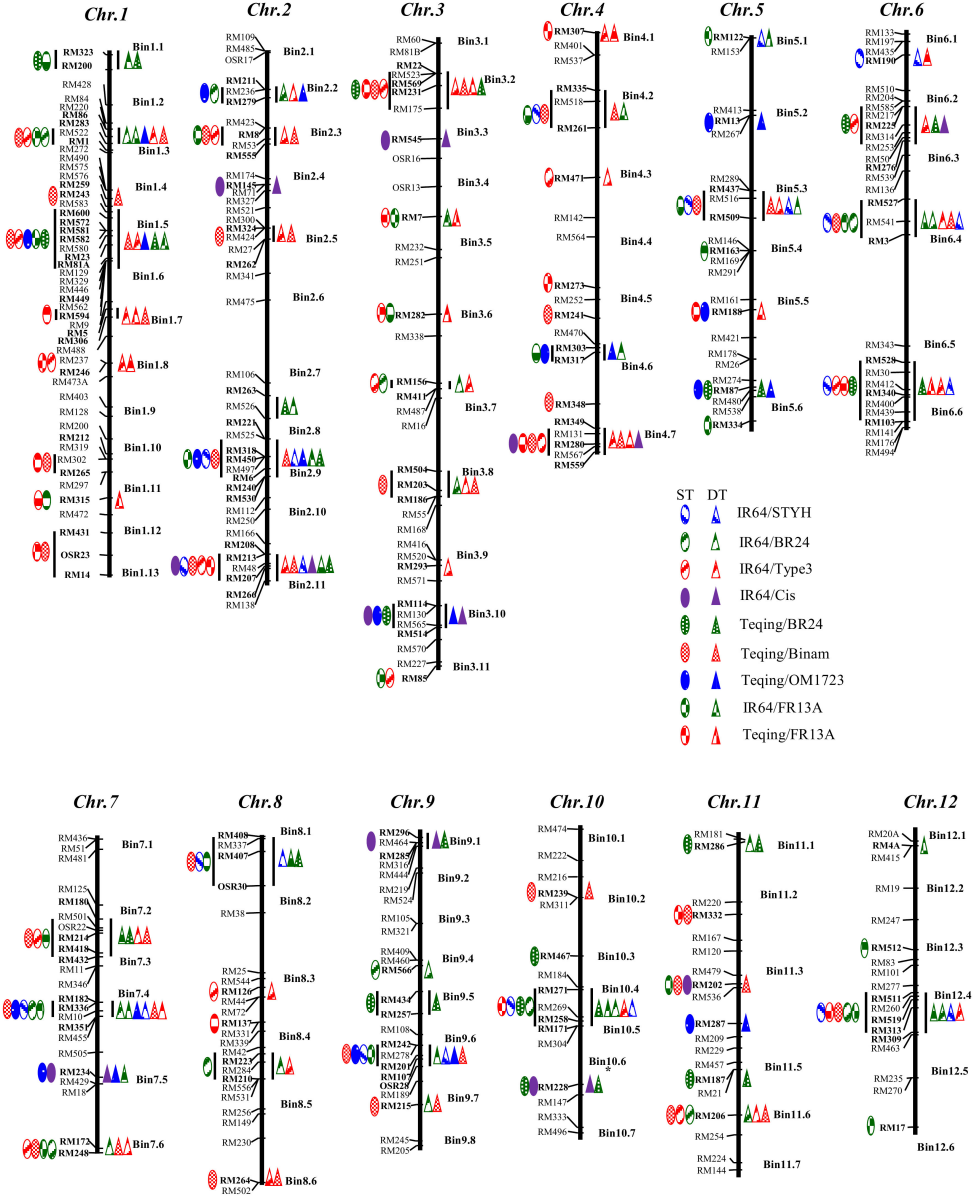
Genomic distribution of 127 loci in 59 genomic regions associated with drought and submergence tolerances (DT and ST) identified in introgression lines selected for DT and ST from nine BC_2_F_2_ populations derived from crosses between two recipients (IR64 and Teqing) and 7 donors based on *χ²* tests of introgression frequencies and linkage disequilibrium analyses (Li et al., 2010; Zhang et al., 2014). Each network contained all DT FGUs (markers and AGs) detected in ILs from a single population/stress case. An AG is defined as a group of unlinked but perfectly and positively associated loci in the ILs from a single population. The hierarchy of each network was determined based on the level of introgression: a single FGU with the highest introgression was put on the top, followed by FGUs with successively decreasing introgression frequencies. The arrowed lines in the networks each has three meanings: (1) the level of introgression of the FGU it pointed; (2) the level of absolute overlap (D’ = 1.00) between the two FGUs it linked; and (3) the inclusive relationship of the FGUs at the lower and upper levels. The number under each marker represents the bin and the one under an AL indicates the number of loci it contains.

DNA was isolated from bulked fresh leaf tissues from all (>30) plants of each BC_2_F_3_ line selected for DT using the CTAB method (Murray and Thompson, 1980) to reconstruct the genotype for each of the original selected BC_2_F_2_ plants. Then, a total of 625 single sequence repeat (SSR) markers across the rice genome (Ware et al., 2002; https://www.gramene.org/) were used to screen the polymorphisms between the recipients and donors, from which 309 SSR markers representing 91 well-distributed bins (each covering a genomic region of 10-20 cM) across the rice genome (**Figure 1**) were used to genotype all drought-selected BC_2_F_3_ ILs. On average, ILs from each BC population was genotyped with 86-158 polymorphic SSR markers that covered >95% of the introgressed donor segments in the ILs.

## Data analyses

### Detection of QTLs for DT in the selected ILs

According to the population genetics theory (Falconer and Mackay, 1996; Hartl and Clark, 1997), identification of QTLs affecting DT (target trait) was performed using the strategy of selective introgression (Zhang et al., 2021a). Specifically, the donor introgression in the 260 BC_2_F_2_ plants initially selected under severe drought from the nine populations were expected to show significant (*P* ≤ 0.001) over-introgression at QTL associated with DT. Thus, *χ²* tests were performed to scan the whole genome to identify marker that showed significant (*P* ≤ 0.001) deviation in genotypic frequencies in the drought BC_2_F_2_ plants of a specific population from the whole genome donor introgression of the population.

### Detection of QTLs for ST

Identification of ST was performed in three steps. First, *χ²* tests were performed to scan the whole genome to identify marker that showed significant (*P* ≤ 0.001) deviation in genotypic frequencies in the DT-ST selected BC_2_F_4_ lines of a specific population from the whole genome donor introgression of the population. Secondly, because those ST BC_2_F_4_ lines were selected from the drought selected BC_2_F_2_ plants, those QTLs showing over-introgression detected by *χ²* tests in the DT-ST selected lines from a BC population were not resulting from selection for ST, but from their high frequencies in the original selected DT plants. To overcome this, we performed a one-tailed conditional *Z* test, $\text{Z}=\frac{OF_{\left(ST\right)}-EF_{\left(ST\right)}}{SD}$ , where $\text{SD}=\sqrt{\frac{\left(OF_{\left(ST\right)})(1-OF_{\left(ST\right)}\right)}{n}}$ , *OF*_(_ *_ST_*_)_ was the observed donor introgression frequency at an identified ST QTL and E*F*_(_ *_ST_*_)_=*OF*_(_ *_ST_*_)_·*si*_(_ *_ST_*_)_ was the expected donor introgression frequency at the ST QTL, *SI_(ST)_* and *n* were the selection intensity and number of the drought selected BC_2_F_2_ plants of the population. Once *Z* ≥ 1.645, i.e. *OF*_(_ *_ST_*_)_ was significantly greater than E*F*_(_ *_ST_*_)_ of the ST QTL at *P* ≤ 0.05, suggesting that this QTL was significantly associated with ST conditional to its frequency in the drought selected BC_2_F_2_ plants. Thirdly, a multi-locus independence probability (MIP) test, *P*_(_ *_AG_*_)_=(*p_i_*)*^rm^* •(1–*p_i_*)*^r^* ^(^ *^n^* ^–^ *^m^* ^)^, was performed to detect individual association groups (AGs) for ST in the DT-ST selected lines of a population. Here, an AG is a group of *r* (*r*≥2) unlinked but perfectly associated loci of equal introgression in the DT-ST selected lines of a population, where *p_i_* is the expected frequency of the donor introgression in the DT-ST selected lines of the BC population, *n* is the number of DT-ST selected lines, *m* is the number of lines that have co-introgression of the donor alleles, and (*n*–*m*) is the number of lines having no introgression at the *r* unlinked QTLs in the AG. Here, (*P_i_*)*^m^* is the probability of *m* lines having co-introgression of the donor alleles and (1-*P_i_*)*^n^* ^-^ *^m^* is the probability of (*n*-*m*) lines having no introgression at *r* unlinked loci. To minimize the false positive rate in detecting AG for ST QTLs, double criteria were used to claim a significant AG, including the *χ²* test for each of the QTLs in an AG should reach *P*< 0.05 and *P_AG_* ≤ 0.001.

### Candidate gene analyses

For important major ST/DT QTLs that were detected in >3 populations, we searched the rice databases (http://rice.uga.edu/; https://www.ricedata.cn/) and identified genes that have reported functions on DT and/or ST and are co-localized with the major QTLs as candidate genes for each of the major ST/DT QTLs. To obtain additional evidence in candidate gene analyses, we performed the gene CDS haplotype analyses (Zhang et al., 2021b) to compare the parental differences at each of the candidate genes to see if the recipient (IR64) allele differed from the donor alleles at each of the candidate genes.

## Results

### Development of ILs with significantly improved ST and/or DT

#### Selection efficiency for DT

Under the DS lowland drought, none of the recipients could produce seeds, but a total of 121 (6.5%) BC_2_F_2_ plants survived the drought stress and produced seeds, ranging from four plants from population IR64/Cis to 23 plants from population IR64/Type3. Under the upland drought, a total of 139 plants (7.4%) survived and produced seeds, ranging from six plants from population Teqing/OM1723 to 27 plants from population IR64/BR24 (**Supplementary Table S1**). Together, a total of 260 BC_2_F_2_ plants survived the drought stresses, including 46 plants form populations IR64/STYH (A), 41 plants from population IR64/BR24 (B), 38 plants from population IR64/Type3 (C), 13 plants from population IR64/Cis (D), 19 plants from population Teqing/BR24 (E), 27 plants from population Teqing/Binam (F), 13 plants from population Teqing/OM1723 (G), 31 plants from population IR64/FR13A (H) and 32 plants from population Teqing/FR13A (I), respectively, with an average selection intensity of 7.3%.

#### Selection efficiency for ST

The 260 BC_2_F_3_ lines selected from the drought stresses in the DS were subjected to the two-week submergence in the WS which killed the recipients, but 125 (48.1%) of the drought selected BC_2_F_3_ lines were able to show >50% recovery rates after the submergence stress. In the replicated confirmation testing for ST under the two-week submergence in the coming DS when the recipients, IR64 and Teqing, were killed by the stress, 124 (47.7%) of the 125 BC_2_F_4_ lines were confirmed to have significantly higher (> 50%) recovery rates, ranging from 10 (31.3%) of the 32 lines from population Teqing/FR13A to 11 (84.6%) of the 13 lines from population IR64/Cis (**Supplementary Table S1**). This result was in contrast to the average recovery rates of 1.08% and 0.85% in 60 IR64 BC_2_F_2_ populations and 57 Teqing BC_2_F_2_ populations screened for ST that included the nine BC_2_F_2_ populations used in this study (Ali et al., 2006; Wang et al., 2015). This result indicated that DT and ST in rice shared a significant proportion of the same genetic controls.

### The genomewide responses to selection for DT

To understand the genome-wide responses of the donor introgressions in the BC progeny to selection for DT, we genotyped the 260 drought selected BC_2_F_3_ lines with 309 polymorphic SSR markers that covered >95% of the introgressed donor segments in the originally selected BC_2_F_2_ plants. The donor introgression patterns in the 260 BC_2_F_2_ plants, characterized by the polymorphic SSR markers across the genome, were reflected in three major ways (**Supplementary Table S1**). First, strong selection under drought resulted in significantly increased donor introgression in the ST ILs. On average, the donor introgression in the 260 BC_2_F_2_ plants, estimated from all polymorphic markers in the resultant BC_2_F_3_ lines, was 0.199 ± 0.052, ranging from 0.139 in the 13 lines of population Teqing/OM1723 to 0.298 in the 38 lines of population IR64/Type3, significantly higher than the Mendelian expectation of 0.125. This excess donor introgression resulted primarily from the excess homozygous donor alleles (0.167 ± 0.043) by 167.2% across the genome at the expenses of greatly reduced heterozygosity by 49.6% in the drought selected plants (**Supplementary Table S1**).

### Identification of QTLs associated with DT

The *χ²* tests using the genotypic data of SSR markers across the rice genome identified 59 genomic regions or QTLs (in 144 cases) that showed significant excess donor introgression in the 260 drought selected lines from the nine BC_2_F_2_ populations (**Figure 1** **;** **Supplementary Table S2**). Of the identified DT QTLs, 13 QTLs were more important and each detected in ≥4 of the populations, while 17 of the DT QTLs were each detected in a single population (**Figure 1**). Specifically, the identified DT QTLs included 11 QTLs on eight rice chromosomes with an average donor frequency of 0.571 in the 46 drought selected BC_2_F_2_ plants of population IR64/STYH (**Supplementary Table S2A**), 14 QTLs on ten chromosomes with an average donor frequency of 0.360 in the 41 drought selected BC_2_F_2_ plants of population IR64/BR24 (**Supplementary Table S2B**), 26 QTLs on ten chromosomes with an average donor frequency of 0.624 in the 38 drought selected BC_2_F_2_ plants of population IR64/Type3 (**Supplementary Table S2C**), nine QTLs on seven chromosomes with an average donor frequency of 0.513 in the 13 drought selected BC_2_F_2_ plants of population IR64/Cis (**Supplementary Table S2D**), 13 QTLs on eight chromosomes with an average donor frequency of 0.484 in the 19 drought selected BC_2_F_2_ plants of population Teqing/BR24 (**Supplementary Table S2E**), 25 QTLs on all chromosomes with an average donor frequency of 0.510 in the 27 drought selected BC_2_F_2_ plants of population Teqing/Binam (**Supplementary Table S2F**), 12 QTLs on eight chromosomes with an average donor introgression of 0.529 in the 13 drought selected BC_2_F_2_ plants of population Teqing/OM1723 (**Supplementary Table S2G**), 21 QTLs on 11 chromosomes with an average donor introgression of 0.422 in the 32 drought selected BC_2_F_2_ plants of population IR64/FR13A (**Supplementary Table S2H**), and 13 QTLs on seven chromosomes with an average donor frequency of 0.408 in the 31 drought selected BC_2_F_2_ plants of population Teqing/FR13A (**Supplementary Table S2I**), respectively. Notably, non-Mendelian segregation distortions with extremely high donor introgression (>0.65) and/or zero heterozygosity were observed in 74 (51.4%) of the 144 cases of DT QTLs identified in specific populations. In particular, 17 of the 26 DT QTLs detected in population IR64/Type3 were of this type.

### Identification of ST QTLs in the DT-ST selected progeny

As shown in **Supplementary Table S1**, 124 of the 260 drought selected BC_2_F_4_ lines from the nine populations had confirmed ST under two rounds of progeny testing under complete submergence for 14 days in the DS and WS. *χ²* tests at individual loci, conditional *Z* tests plus the multi-locus independence probability (MIP) tests using the genotypic data of SSR markers of the 124 DT-ST selected BC_2_F_4_ ILs from the nine populations identified 68 QTLs (in 145 cases) associated with ST (**Figure 1** and **Supplementary Table S2**). Specifically, the identified ST loci included 10 QTLs with an average donor frequency (F_(donor)_) of 0.545 in the 26 ST BC_2_F_4_ lines of population IR64/STYH (**Supplementary Table S2A**), 10 QTLs with an average donor frequency of 0.431 in the 25 ST BC_2_F_4_ lines of population IR64/BR24 (**Supplementary Table S2B**), 16 QTLs on eight chromosomes with an average F_(donor)_ of 0.642 in the 20 BC_2_F_4_ lines of population IR64/Type3 (**Supplementary Table S2C**), nine QTLs on seven chromosomes with an average F_(donor)_ of 0.533 in the 11 ST BC_2_F_4_ lines of population IR64/Cis (**Supplementary Table S2D**), 10 QTLs plus two AGs on seven chromosomes with an average F_(donor)_ of 0.478 in the eight ST BC_2_F_4_ lines of population Teqing/BR24 (**Supplementary Table S2E**), 11 QTLs and seven AGs on all 12 chromosomes with an average F_(donor)_ of 0.455 in the 11 ST BC_2_F_4_ lines of population Teqing/Binam (**Supplementary Table S2F**), 12 QTLs on eight chromosomes with an average F_(donor)_ of 0.574 in the nine BC_2_F_4_ lines of population Teqing/OM1723 (**Supplementary Table S2G**), 14 QTLs and five AGs on 11 chromosomes with an average F_(donor)_ of 0.355 in the 10 ST BC_2_F_4_ lines of population IR64/FR13A (**Supplementary Table S2H**), and 12 QTLs and three AGs with an average F_(donor)_ of 0.397 in the 13 ST BC_2_F_4_ lines of population Teqing/FR13A (**Supplementary Table S2I**), respectively. Notably, non-Mendelian segregation distortions with extremely high donor introgression (>0.65) and/or zero heterozygosity were observed in 97 (67.4%) of the 144 cases of ST loci identified in specific populations.

### Genetic overlap between DT and ST

**Figure 2:**
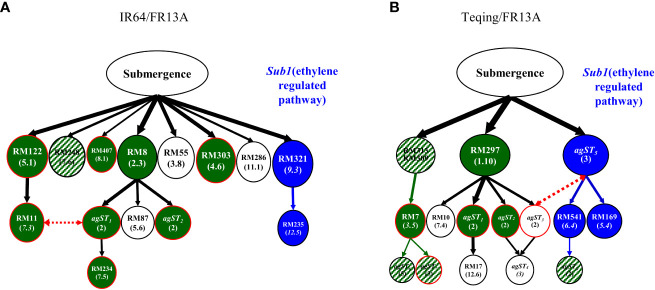
Genetic overlap between drought tolerance (DT) and submergence tolerance (ST) revealed in genetic networks (multi-locus structures) constructed from ST loci from the two BC_2_F_2_ populations, in which each oval represents a locus or association group (AG) identified in 14 BC_2_F_2_ plants selected under submergence stress from population IR64/FR13A//IR64 **(A)** and from eight BC_2_F_2_ plants of population Teqing/FR13A//Teqing **(B)** (Wang et al., 2015). Those loci in green color were detected as ST loci identified in the ten BC_2_F_4_ lines selected first for DT and then for ST from population A and in 13 BC_2_F_4_ lines selected for DT-ST from population B (**Table S3**), respectively, while those ovals in patched green represent loci detected with closely linked markers of the same bins. Those ovals with red borderlines were DT loci identified in the same populations, while those ovals in blue and while colors were not detected in the DT-ST lines in the two populations.

When compared the DT and ST QTLs identified in the same populations, we observed an average 55.4% of the genetic overlap (the shared proportion of the same QTLs for DT and ST identified in the same populations), close to the mean 50.9% of the phenotypic overlap between drought selected and ST BC_2_F_4_ lines, ranging from 29.2% in population Teqing/FR13A to 80% in population IR64/Cis (**Figure 1** and **Supplementary Table S2**). In fact, the genetic overlap was positively correlated with the phenotypic overlap (*r* = 0.716) across the BC populations (**Supplementary Table S1**). To better illustrate the genetic overlap between rice DT and ST, we compared the genetic mapping results from two separate experiments using the same two BC_2_F_2_ populations between recipients (IR64 and Teqing) and donor FR13A. **Figure 2A** shows the ST genetic network (multi-locus structure) containing 12 loci and two AGs identified in 14 surviving plants selected under submergence stress from the IR64/FR13A BC_2_F_2_ population (Wang et al., 2015), of which nine QTLs were also detected in the ten DT-ST selected BC_2_F_4_ lines of the same population in this study (**Supplementary Table S2H**). Moreover, eight of the nine ST QTLs detected in the ST BC_2_F_2_ plants were also associated with DT in the 32 drought selected BC_2_F_2_ plants from the same population. Only five ST QTLs identified in the previously submergence selected BC_2_F_2_ plants were not detected in the ten DT-ST BC_2_F_4_ lines. Similarly, the genetic network detected in the eight submergence-surviving plants of the Teqing/FR13A BC_2_F_2_ population containing 6 QTLs and eight AGs (**Figure 2B**, Wang et al., 2015), ten of which were also detected as ST QTLs in the 13 DT-ST BC_2_F_4_ lines of the same population (**Supplementary Table S2I**). Moreover, four of the ST QTLs detected in the ST BC_2_F_2_ plants were associated with DT in the 31 drought selected BC_2_F_2_ plants from the same population. Ten ST QTLs identified in the eight submergence selected BC_2_F_2_ plants were not detected in the ten DT-ST BC_2_F_4_ lines in the Teqing/FR13A BC_2_F_2_ population (**Figure 2B**). Strikingly, in both populations, the ST loci detected in submergence-selected BC_2_F_2_ plants but not in the DT-ST selected BC_2_F_4_ lines of the same populations shared a common branch of associated QTLs (the blue colored loci of **Figure 2**), in which one QTL near bin 9.3 in the upper regulatory position of the branch harbors the well-known *Sub1* gene for ST (Xu et al., 2006). Apparently, the *Sub1A* (an ethylene response transcriptional factor) and its regulated loci were eliminated in the drought-selected plants of both IR64/FR13A and Teqing/FR13A BC_2_F_2_ populations, but in over-introgression in the ST selected BC progenies of the same two populations (Wang et al., 2015).

### Candidate genes for major QTLs affecting DT and ST

Among the identified QTLs, we found 16 QTLs for either DT and/or ST were co-localized near the same markers or in the same bin regions in three or more of the populations simultaneously (**Table S3**). These hotspots or clustered QTLs were definitively designated as the major QTLs. These included seven QTLs (*qDT1.7*, *qDT2.2*, *qDT3.8*, *qDT7.5*, *qDT7.6*, *qDT10.5*, and *qDT11.6*) for DT each detected in three populations, six QTLs (*qDT2.9*, *qDT3.2*, *qDT4.7*, *qDT5.3*, *qDT9.6*, and *qDT12.4*) each identified in four populations, two QTLs (*qDT1.3* and *qDT7.4*) detected in five populations, and one QTL (*qDT2.11*) identified in six populations. Eleven major QTLs were found to affect ST, including three (*qST2.9*, *qST5.3*, and *qST6.5*) and six QTLs (*qST1.3*, *qST3.2*, *qST4.7*, *qST7.4*, *qST7.6*, and *qST9.6*) identified in three and four populations, respectively. Two QTLs (*qST2.11* and *qST12.4*) were each identified in five populations simultaneously (**Table S3**).

**Table S4** shows the eight most likely candidate genes for the identified major QTLs and their population genetics parameters in different rice populations. The first one is *LOC_Os01g09620* co-localizing with *qST1.3*/*qDT1.3* and encoding a CCCH-type zinc finger protein to have the function for DT and is responsive to ethylene (Kong et al., 2006.). At this locus, IR64 has Hap2 for susceptibility to both drought and submergence, while the three donors (BR24, Type3, and OM1723) have Hap1 contributing DT/ST. In the *qDT2.11*/*qST2.11* region, the most likely candidate gene was *LOC_Os02g57530* encoding an ethylene receptor which was potentially to be associated with ST and DT (Yau et al., 2004). At this locus, IR64 has Hap2 for susceptibility to both drought and submergence, while six donors contributing tolerance have Hap1, Hap3, Hap4, and Hap5. We identified *LOC_Os02g48010* as the most likely candidate for *qDT2.9*/*qST2.9*. It encodes a nuclear matrix constituent protein 1-like, and IR64 has Hap3 while three donors (BR24, Type3, and OM1723) have Hap1, Hap2, and Hap4 contributing DT/ST. The most likely candidate gene for *qDT2.9*/*qST2.9* is *LOC_Os04g54474* encoding a bZIP transcription factor, and its homologous gene (*OsbZIP86*) is associated with DT/ST (Nijhawan et al., 2008; Gao et al., 2022). The gcHaps of the DT/ST contributing donors (Type3, Cisanggarung, and Binam) have either Hap1 or Hap3, while IR64 has Hap2. For *qDT5.3*/*qST5.3*, the most likely candidate gene is *LOC_Os05g28350* encoding an AP2 domain containing protein. The evidence was from the allelic differences between IR64 (Hap3) and the donors (Hap2, Hap4, and Hap5) contributing to DT/ST. For *qDT7.6*/*qST7.6*, the most likely candidate gene is *LOC_Os07g48630* encoding a protein responsive to ethylene stimulus (Mao et al., 2006) with obvious allelic differences between IR64 (Hap4) and the donors (Hap1, Hap6, and Hap7) contributing to DT/ST. The most likely candidate gene for *qDT9.6*/*qST9.6* is *LOC_Os09g35010*, encoding AP2/EREBP transcription factor which is reportedly associated with DT (Figureueiredo et al., 2012) with obvious allelic differences between IR64 (Hap1) and the donors (Hap2 and Hap3) contributing to DT/ST. As the most likely candidate gene for *qDT12.4*/*qST12.4*, *LOC_Os12g35610* encodes an ethylene-induced protein functioning in root aerenchyma formation under oxygen-deficient condition (Liu et al., 2012) with obvious allelic differences between IR64 (Hap3) and the donors (Hap2) contributing to DT/ST (**Table S4**). Except for *LOC_Os02g48010* which is a conserved gene with a low diversity (*E_H_* = 0.09) and only two gcHaps in rice populations, the remaining seven candidate genes were high diverse (*E_H_* is between 0.42 and 1.18, and gcHapN is between 4 and 11) within and among different rice populations (**Table S4**).

## Discussion

In most rice breeding programs of Asia, breeding for improved DT and ST has been largely separate efforts using the conventional pedigree breeding approach involving line crossing and phenotypic selection using relatively few landraces with either high DT or ST as donors. Meanwhile, few efforts have been taken to simultaneously improve DT and ST, even high yield rice varieties with good DT and ST are desperately needed in many rainfed areas of South Asia. The primary reason appeared to be the “absence” of germplasm accessions with excellent DT and ST to be used as donors in crossing schemes of breeding programs, and a partial reason was the apparent negative phenotypic association between DT and ST observed in some rice accessions with either high levels of DT or ST. As an attempt for simultaneous improvement and genetic dissection of DT and ST in rice, we demonstrated here that both reasons mentioned above were apparently based on misconceptions. In this study, none of the donors of our BC populations had good levels of DT and ST, though OM1723 has good DT (Lafitte et al., 2006) and FR13A has excellent ST. Nevertheless, under the severe drought and submergence stresses adopted in our drought and submergence screening that killed the recipients, IR64 and Teqing, we were able to achieve average selection intensities of 7.3% and ~50% in our first round selection for DT and the second round selection for ST, resulting in the development of 124 BC_2_F_4_ lines with significantly improved DT and ST. This indicated that all donors contain many genes/alleles for improved DT and ST. Similar results were obtained in our BC breeding effort for developing green super rice with improved tolerances to multiple abiotic stresses such as drought, salt, and submergence (Ali et al., 2017). Thus, the empirical view of “absence’ of donors for improving multiple abiotic stress tolerances was a misconception. Meanwhile, by characterizing the donor introgression patterns in the 260 drought selected and 124 DT-ST selected BC progenies, we were able to identify 144 cases of 59 DT QTLs and 145 cases of 68 ST QTLs with an average 55% of the identified QTLs associated with both DT and ST (**Supplementary Table S2**). In particular, all 68 ST QTLs identified in this study were also detected in the submergence-selected ILs from 12 BC populations (Wang et al., 2015) Moreover, a comparison of the DT QTLs identified in this study with previously cloned DT genes revealed that 79.2% QTLs (n = 114) of the DT QTLs colocalized in the same or overlapping regions that harbor genes known to be associated with DT (**Supplementary Table S2**). For example, *OsJAZ1* (LOC_Os04g55920) encoding a Jasmonate ZIM-domain protein and *SNAC3* encoding a NAC (NAM, ATAF1/2, and CUC2) protein were reported to play important roles in rice tolerances to abiotic stresses (drought, salinity, and cold) (Fang et al., 2015; Fu et al., 2017). Here, we demonstrated that the strategy of SI was powerful and efficient not only for simultaneous improvement and genetic dissection of rice tolerances to single abiotic stresses, as reported previously (Zhang et al., 2014; Wang et al., 2015; Cui et al., 2018; Liang et al., 2018), but also for simultaneous improvement and genetic dissection of both DT and ST in rice. This was not surprising considering the original population size of 400+ individuals for each of the BC populations to be screened for DT and the number/diversity of the parents involved in the developed populations. Thus, the genomewide introgression patterns in the drought- and DT-ST selected BC progenies revealed several genetic mechanisms underlying rice DT and/or ST that worth further discussion.

In this study, we observed two related respects regarding the donor introgression of the BC progenies in response to the phenotypic selection for DT: 1) large numbers of QTLs showing excess donor over-introgression and 2) ~50% of these DT QTLs showing extreme non-Mendelian or ‘epigenetic’ segregation with very high donor introgression and/or loss of heterozygosity (LOH) that was responsible for the observed whole genome reduction in heterozygosity by ~50% in the drought selected BC_2_F_2_ plants. Moreover, QTLs showing ‘epigenetic’ segregation and LOH appeared to be randomly distributed across the genome. While the over-introgression at loci with donor alleles associated with DT was expected theoretically (Falconer and Mackay, 1996; Hartl and Clark, 1997), LOH at most DT QTLs was a puzzling observation. In fact, similar epigenetic segregation for greatly reduced heterozygosity was also observed in the BC progenies in different genetic backgrounds selected under drought (Cui et al., 2018), submergence (Wang et al., 2015), salinity (Li et al., 2017a) and multiple stresses (Ali et al., 2017). Moreover, the slat-selected F_3_ progeny derived from several F_2_ populations of crosses between the drought-selected ILs, when segregating at >20 large genomic segments, showed genomewide LOH (~98% homozygosity) and some of the ‘epigenetic’ regions were inherited from donor DT-QTLs showing LOH, indicating they were transgenerational (Li et al., 2017a). Unfortunately, the genetic and molecular mechanism(s) underlying this type of stress-induced LOH remain largely unknown and should be investigated in future. Also, the observed LOH in the DT QTLs and genomewide reduced heterozygosity of the DT selected ILs made it impossible to distinguish which of the detected DT/ST QTLs were dominant ones, even though some of them might be dominant ones.

Our results suggested the presence of three genetic mechanisms underlying relationships between DT and ST in rice: 1) QTLs with pleiotropic effects on both DT and ST; 2) QTLs with opposite effects on both DT and ST; and 3) QTLs with independent effects on DT and ST. The first one was evidenced by an average ~55% overlap between the identified DT and ST QTLs, which explained well the observed average ~50% of the drought selected BC progeny showing significantly improved ST. This observation was in contrast to the average of 7.3% SI for DT in the nine BC population and an average<1% SI for ST from 117 random BC_2_F_2_ populations in the same genetic backgrounds (Ali et al., 2006; Wang et al., 2015). Additional evidence came from our candidate gene analysis in which eight of the 16 major QTLs were associated with both DT and ST. Moreover, we were able to identify one most likely candidate gene with known functions on DT or ST for each of the eight major DT/ST QTLs (**Table S4**). Secondly, while all 68 ST QTLs identified in the DT-ST selected BC progeny of the nine populations were also discovered in the ST selected BC progeny previously (Wang et al., 2015), a group of highly associated ST QTLs previously detected in ten of the 12 random BC_2_/BC_3_ populations from crosses between three recipients (IR64, Teqing and NPT) and three donors (TKM9, BR24 and FR13A) (Wang et al., 2015), were virtually completely eliminated in all drought selected BC progenies of this study. Detailed comparisons between results from the two separate studies (**Figure 2**) indicated that this group of ST loci were those involved in the *Sub1* (an ethylene responsive transcription factor) regulated pathway. Moreover, ~50% of the DT loci identified in the drought selected progeny were not associated with ST in this study, most of which co-localize with DT-ST loci in this study and were negatively associated with loci in the *Sub1* regulated pathway reported previously (Wang et al., 2015). Additional evidence came from the fact that the donor alleles at the *Sub1* locus near bin 9.3 and most of its associated ST loci identified in the submergence selected ILs from five additional BC_2_ and BC_3_ populations with the same genetic backgrounds (IR64 and Teqing) were all eliminated in the DT selected ILs of this study (Wang et al., 2015). This negative association between the *Sub1* regulated pathway/loci for ST and loci for DT appeared to explain the negative phenotypic relationship between the high level ST and poor DT of FR13A because the ST of FR13A is known to be controlled by the *Sub1* regulated pathway (Xu et al., 2006; Kudahettige et al., 2011). Thirdly, of the total 289 cases of the DT and ST QTLs identified in the nine populations, 42 cases of DT QTLs and 87 cases of the ST QTLs appeared to be genetically independent from one another. Thus, our results were consistent with the current knowledge that either DT or ST in rice are controlled by multiple signaling pathways mediated by different phytohormones such as abscisic acid (ABA), jasmonates (JAs), salicylic acid (SA), ethylene (ET) and there are complex cross-talks between or among different phytohormone-mediated signaling pathways which remain poorly understood (Fang et al., 2008; Fukao et al., 2011; Li et al., 2017b). In this regard, our results raised two interesting questions that may help to understand the cross-talks between different signaling pathways underlying DT and ST in rice. Firstly, given the presence of antagonistic interaction between ABA and ethylene in their biosynthesis and their mediated signaling pathways in response to drought in rice and other plants (Yang et al., 2007; Deb et al., 2016), it would be of great interest to validate if the elimination of the *Sub1* (ethylene) mediated ST loci/pathway in the drought selected rice progenies resulted from the introgression of donor alleles acting in the ABA mediated signaling pathway(s) for DT. In other words, is the antagonistic interaction between the ABA and ethylene mediated signaling pathways responsible for the negative association for DT and ST in rice? and if so, how is it achieved at the molecular level? Secondly, what is the molecular basis for the positive relationship between DT and ST in rice observed in this study? In this respect, the ILs with contrasting phenotypes and different combinations of genetic loci for DT and/or ST developed in this study provided valuable materials for future research to answer the questions by resolving multiple candidate genes/pathways using different combinations of different genetic/molecular/omic tools.

The genetic bases of DT and ST discussed above have several implications on how to increase the selection efficiencies and effectiveness for improved DT/ST in rice breeding. First, most rice varieties/accessions can be ‘good’ donors in a BC breeding program for improving DT and ST as long as the donors are not closely related to recipients, because most DT loci are either pleiotropic or independent in controlling DT and ST. Second, our results strongly suggest that the *Sub1* regulated pathway for ST should not be used when both DT and ST are the breeding targets, because of the strong negative association between the *Sub1* regulated pathway and DT observed in this study. It should be pointed out that in real breeding efforts for improving DT and ST, DT should be the target in the initial single plant selection because DT apparently had higher heritability than ST. Thus, more plants would be selected in the initial screen for DT so that the selected progeny from each segregating population would have a large population size to allow exploitation of the residual diversity in ST and other target traits such as yield potential, grain quality and resistance biotic stresses. The SNPs between IR64 and the donors within the gene CDS regions of the eight candidate genes for the major DT/ST QTLs can serve as powerful molecular markers in MAS for simultaneous improvement of DT and ST in rice. Also, the genomewide LOH in the drought selected progeny is expected to speed up the breeding process by quicker genetic fixation of the selected lines. The key for success in breeding DT and ST rice varieties is how to manage strong and appropriate levels of drought and submergence stresses in screening segregating progenies during breeding. Finally, we have again demonstrated the superior power and efficiency of selective introgression not only in simultaneous improvement and genetic dissection of single complex traits but also of two or more complex traits, which can relatively easily be adopted by small breeding programs in developing countries to develop varieties adapted to different rice ecosystems.

## Conclusion

Our results indicated that BC breeding and phenotypic selection were highly effective for simultaneous improvement of DT and/or ST in rice. Genetic tracking and characterization of donor introgression in the 260 selected ILs using markers revealed three important aspects of the genetic bases of DT and ST in rice: (1) significant over-introgression of donor alleles at 59 DT QTLs and 68 ST QTLs with an average 55% of the identified loci associated with both DT and ST; (2) approximately half of the DT QTLs exhibited a ‘epigenetic’ segregation with very high donor introgression and/or loss of heterozygosity, and (3) three different genetic mechanisms (the loci with pleiotropic, opposite and independent effects on DT and ST) underlying the relationship between DT and ST. The developed DT, ST ILs, and their genetic information on the donor alleles for DT and ST would provide a useful platform for developing superior DT and ST varieties through breeding by design in future.

## Data availability statement

The original contributions presented in the study are included in the article/**Supplementary Material**. Further inquiries can be directed to the corresponding authors.

## Author contributions

ZL and JA designed and conceived the experiments. WW, RL and JD performed the phenotypic screening and evaluation. CZ, ML, TF, YL, FW, WZ, EL performed analysis and interpretation of the data. ZC, LM, and WW drafted the manuscript. ZL, TZ, BF, JX and FZ revised the MS. All authors contributed to the article and approved the submitted version.
